# Epidemiology of treatment resistant depression among major depressive disorder patients in Israel

**DOI:** 10.1186/s12888-022-04184-8

**Published:** 2022-08-11

**Authors:** Sarah Sharman Moser, Gabriel Chodick, Shulamit Gelerstein, Nava Barit Ben David, Varda Shalev, Orit Stein-Reisner

**Affiliations:** 1 Maccabi Institute for Research and Innovation (Maccabitech), Maccabi Healthcare Services, Tel Aviv, Israel; 2 Sackler Faculty of Medicine, Tel Aviv University, Tel Aviv, Israel; 3 Janssen Israel, J-C Health Care Ltd, Kibbutz Shefayim, Tel Aviv, Israel

**Keywords:** Major depressive disorder, Treatment resistant depression, Real-world retrospective database study

## Abstract

**Introduction:**

Major depressive disorder (MDD) is one of the most common mental disorders worldwide, estimated to affect 10–15% of the population per year. Treatment resistant depression (TRD) is estimated to affect a third of these patients who show difficulties in social and occupational function, decline of physical health, suicidal thoughts and increased health care utilization. We describe the prevalence of MDD, TRD and associated healthcare resource utilization in Maccabi Healthcare Services (MHS), a 2.5 million-member state-mandated health service in Israel.

**Methods:**

All MHS members with an MDD diagnosis were identified within the years 2017–2018 and prevalence assessed by age, sex and TRD. To assess the incidence of MDD, members aged 18–65 years at the start of any MDD episode were identified between 1^st^ January 2016 and 31^st^ May 2018 with at least one systemic first-line antidepressant treatment within three months before or after the initial episode. Treatment patterns, time on first-line treatment, and healthcare resource utilization were compared by TRD.

**Results:**

A total of 4960 eligible MDD patients were identified (median age = 51 years, 65% female), representing a period prevalence of 0.218%, and of those, a high proportion of patients received drug treatment (92%). Among incident MDD cases (*n* = 2553), 24.4% had TRD. Factors associated with TRD included increasing age and personality disorder. Median time on treatment was 3.7 months (longer for those without TRD than those with) and 81.9% of patients purchased more than one month’s supply of therapy. In the year after index, patients with TRD had a significant increased number of visits to primary care physicians, psychiatrists, emergency room visits, general hospitalizations, and psychiatric hospitalizations.

**Conclusion:**

Our study shows that prevalence of MDD in Israel is low compared to other countries, however once diagnosed, patients' are likely to receive drug treatment. Among patients diagnosed with MDD, the proportion of TRD is similar to other countries, increases with age and is associated with increased healthcare utilization, therefore should be a focus of continued research for finding effective long term treatment options.

## Introduction

Clinical depression or Major Depressive Disorder (MDD) is one of the most common mental disorders worldwide, accounting for 4.4% of the disease burden worldwide and 7.2% in the European Union [1–3]. Prevalence rates vary by age, with women more commonly affected than men [4, 5] and lifetime rates varying by country, between 1% in the Czech Republic and 16.9% in the United States of America [2]. MDD has substantial impact on overall functioning and quality of life, associated with high comorbidity [6] and a high burden upon healthcare services [7]. By the year 2020, depression was second in the ranking of Disability Adjusted Life Years (DALY) calculated for all ages. MDD has a chronic or recurrent course, characterized by depressive episodes that can last on average for a year, but can also cause disability between episodes [8].

Clinical guidelines recommend treatment with an antidepressant medication for 6–12 weeks in the initial acute phase for a first episode and 4–9 months of continued treatment after this period. Patients may require further maintenance therapy and long term management, switching to a different drug or combination therapy depending on response and severity of the depressive episode [9, 10].

It is estimated that 30%–40% of patients with MDD do not respond to typical antidepressant medications [11], showing treatment resistant symptoms and failure to achieve remission, with difficulties in social and occupational function, decline of physical and mental health, suicidal thoughts [12] and lower quality of life [1, 7, 12]. Treatment resistant depression (TRD) is associated with increased health care utilization and cost with at least 12% more outpatient visits, increased use of psychotropic medications and double the risk of hospitalization than other patients suffering from MDD [13].

There is no definitive definition for TRD. The most common definition requires a minimum of two prior treatment failures for adequate dose and adequate duration in a current episode [14–16], and further defined as a failure to respond to two adequate trials of different antidepressants given for 6–8 weeks at adequate doses [17, 18]. Other definitions exist including failure to achieve remission to at least one, three or five antidepressant drugs [19–21], or a staging system which includes failure of different numbers of antidepressant drugs and electroconvulsive therapy (ECT) [1, 15, 22].TRD prevalence estimates vary widely dependent on the definition used, from 35% in a study limited to subjects with a MDD diagnosis [23] to less than 10% in a study that included a wider range of depression diagnoses among subjects [24]. A real world study in primary care found a prevalence rate of 22% among 1212 patients with MDD [25]. Studies have shown that TRD response rates are poor, with one study showing a 10% one-year response rate to standard MDD treatments [26]. Other therapies that can be tried include ECT, repetitive transcranial magnetic stimulation, intravenous/intranasal ketamine, inhaled nitrous oxide, vagus nerve stimulation, deep brain stimulation, magnetic seizure therapy and buprenorphine, and also psychosocial and cultural therapies [1].

We describe here a retrospective cohort study of the epidemiology, characteristics, treatment patterns and healthcare resource utilization of patients with MDD in Israel.

## Methods

### Data source

This retrospective cohort study was conducted using the computerized databases of Maccabi Healthcare Services (MHS), a state-mandated insurer-provider with 2.5 million members, representing a quarter of the population in Israel, and shares similar sociodemographic characteristics with the general population [27]. The MHS database contains longitudinal data that are automatically collected since 1993 for a stable population people (with less than 1% of members moving out each year), including diagnosis data, laboratory results from a single central laboratory, pharmacy prescription and purchase data, hospitalizations, procedures and consultations. MHS uses the *International Classification of Diseases, Ninth Revision, Clinical Modification* (ICD-9-CM) coding systems, as well as self-developed coding systems to provide more granular diagnostic information. Procedures are coded using *Current Procedural Terminology* (CPT) codes.

### Study population

Two separate study cohorts were analyzed in this study:

#### Period prevalence cohort

In this retrospective cohort study the period prevalence was assessed among all MHS members with at least one MDD ICD-9-CM diagnosis code (296.2, 296.3 or 296.35) from a psychiatrist or general physician for the period 2017–2018 (to allow for an episode of up to a year). This cohort consisted of all patients with a diagnosis code, whether they received treatment or not.

#### Incidence cohort

We identified MHS members aged 18 to 65 years with the start of any MDD episode (main study cohort) between 1^st^ January 2016 and 31^st^ May 2018 (with a minimum of one year of follow up). The start of a `MDD episode was defined as an ICD-9-CM diagnosis code in the medical notes with a gap of at least one year to a previous diagnosis [28]. To be included in this incident study cohort, patients had to have received at least one systemic first-line (L1) therapy for MDD. Index date was set as the date of L1 antidepressant treatment initiation within 3 months before or after MDD episode start date. Patients with less than one year of healthcare registration in MHS or a diagnosis of schizophrenia or bipolar disease before index date were excluded.

A sub-analysis was performed for patients with the same inclusion/exclusion criteria, but for those with a first ever recorded MDD episode within the MHS system.

### Study variables

Demographic and clinical data collected included age at index date, sex, socioeconomic status (SES), residence area, prevalence of comorbid conditions, body mass index (BMI) and smoking. SES was categorized into quartiles based on the poverty index of the member’s enumeration area, as defined by 2008 National Census [29]. The poverty index is based on several parameters including, household income, educational level, crowding, physical conditions, and car ownership. Smoking data were collected from physician reporting and classified into ever, never or unknown.

Baseline chronic diseases were identified using validated MHS registries, (for diabetes mellitus [30], hypertension [31], chronic obstructive pulmonary disease [COPD], cardiovascular disease [32], hypertension, osteoporosis [33], cancer [34]) or by two or more ICD-9-CM diagnosis codes before index date on separate physician appointments for postpartum depression, anxiety, panic disorder, personality disorder and social phobia. The registries were developed in order to improve the quality of chronic care delivery to its members and are continuously updated, and identify patients via automatic search formulas, as opposed to being dependent upon active reporting by physicians. Cancer history was obtained from the National Cancer Registry which uses diagnoses linked to pathology reports and cross referenced with cancer medication approvals in MHS. In addition, comorbidity was measured by the Deyo-Charlson Comorbidity Index [35] and augmented using the MHS chronic disease registries. All comorbidities were measured in the one-year pre-index period. Healthcare services utilization included primary care physician (PCP) visits, hospitalizations (number and duration), emergency room (ER) visits and ECT therapy.

### Treatment patterns

Treatment lines (L1-3) were defined at the patient level according to the sequence of dispensed medication. Antidepressant drugs were grouped into selective serotonin reuptake inhibitors (SSRI) monotherapy, other drug monotherapy (serotonin and noradrenaline reuptake inhibitors; monoamine oxidase inhibitors; atypical antidepressants including venlafaxine, duloxetine, vortioxetine, bupropion, mirtazapine, milnacipran), combination therapy (any combination of at least two medications) and tricyclic antidepressants (TCA) monotherapy. Addition of a new drug to a current regimen was considered a new treatment line, and cessation of a medication from a combination regimen (likely due to tolerance issues) was considered the same line.

TRD was defined by purchase of at least three lines of treatment within the first 12 months after index date. Type of L1 antidepressant use within the MDD cohort and time on treatment were determined.

### Statistical analysis

Descriptive analyses were conducted to compare the demographic, clinical and treatment characteristics for the study cohort for those with and without TRD in the one-year period following index date. Categorical variables were reported as frequency and percentage and compared using chi-square testing, and continuous variables were reported as mean (standard deviation [SD]) or median (interquartile range [IQR]) and compared using the t-test. 

Backward logistic regression was used to compute adjusted odds ratios and 95% confidence intervals (CI) for the explanatory variables for factors associated with TRD.

Treatment duration was assessed using Kaplan–Meier analysis, and median time on treatment with 95% CI presented. Discontinuation was defined as at least 90 days' survival after run out of last treatment or line switch.

All analyses were conducted using IBM SPSS Statistics for Windows, Version 22.0. Armonk, NY: IBM Corp, or R version 3.5.1, and a *P* value < 0.05 was considered statistically significant.

The study was approved by the local ethics review board of MHS in Israel.

## Results

### Period prevalence cohort

**Fig. 1:**
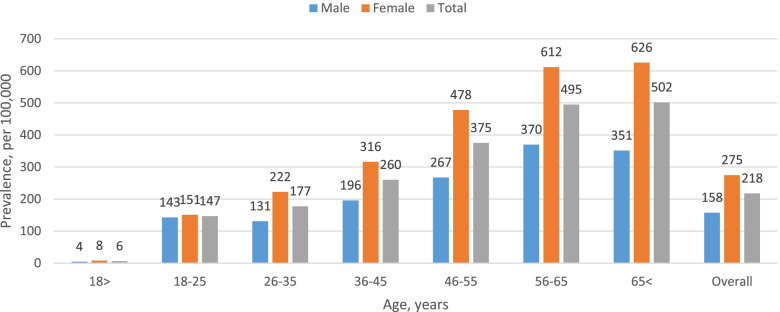
Age-specific period prevalence of patients with major depressive disorder by sex for the period 2017–2018, per 100,000 population, *n* = 4960

A total of 4960 patients had a prevalent MDD episode in the prevalence period (2018). The number of patients with MDD increased with age (median age was 51 years, IQR 38–63) and 65% were female (Fig. 1). Overall prevalence of MDD in MHS was 0.218%. When weight adjusted according to the WHO standard world population, prevalence was 0.202% (data not shown).

**Fig. 2:**
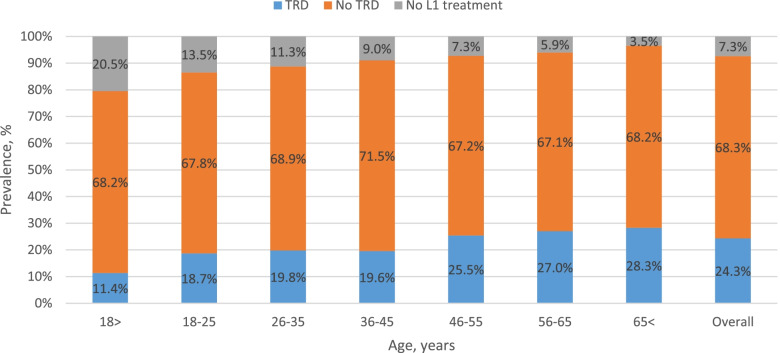
Age-specific period prevalence of all patients with major depressive disorder by treatment resistant depression for the period 2017–2018, by relative distribution of the prevalent cohort, *n* = 4960. TRD, treatment resistant depression; L1, first-line treatment

**Fig. 3:**
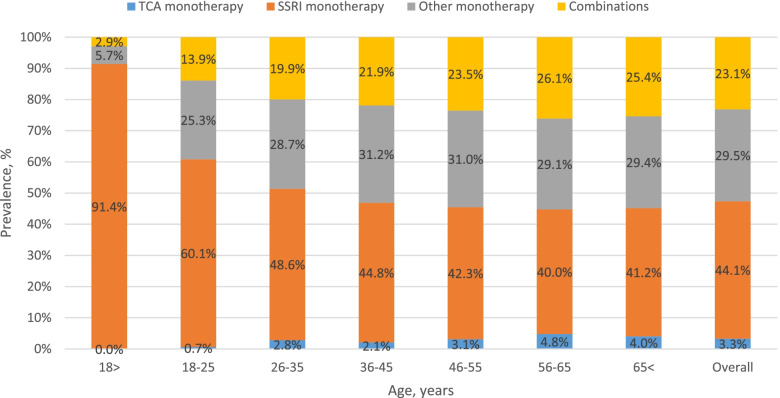
Age-specific period prevalence of patients with major depressive disorder by first-line treatment for those who received treatment for the period 2017–2018, by relative distribution of the prevalent cohort, *n* = 4596. TCA, tricyclic antidepressants; SSRI, selective serotonin reuptake inhibitors

Among those diagnosed, mean TRD was 24.3%, and the proportion of the cohort with TRD increased with age (11.4% for age < 18 years to 28.3% for age > 65 years, Fig. 2). In addition, 92.7% of patients were treated with L1 therapy. Figure 3 shows distribution of L1 treatment by age, with SSRI being the drug of choice for under 18 year olds (91.4%), with use declining to 41.2% for patients over the age of 65 years.

### Incidence cohort

A total of 2553 patients had a new MDD episode in the study period (1/1/2016–31/5/2018) and initiated drug treatment. Of these, 24.4% had TRD according to the definition we used, and 68.1% had a first ever recorded MDD episode.

Median age at start of treatment was 47 years (IQR 36–56), 64.4% were female, 21.6% had hypertension, 11.1% had diabetes, 24.4% had anxiety, 6.1% had personality disorder and 48.9% were past or present smokers (Table 1).

**Table 1:** Demographic and clinical characteristics of the incident study cohort (patients with the start of any major depressive disorder episode within the study period, 1/1/2016–31/5/2018) at treatment initiation, *n* = 2553

Patient characteristics		Patients without TRD (*N* = 1929, 75.6%)	Patients with TRD (*N* = 624, 24.4%)	Total (*N* = 2553)	*P*-value
Age, y	Median (IQR)	46 (35, 55)	49 (38, 56)	47 (36, 56)	0.001
Sex	Male	672 (34.8%)	237 (38.0%)	909 (35.6%)	0.154
	Female	1257 (65.2%)	387 (62.0%)	1644 (64.4%)	
Socio-economic status	Low	581 (30.1%)	188 (30.1%)	769 (30.1%)	0.711
	Medium	402 (20.8%)	121 (19.4%)	523 (20.5%)	
	High	946 (49.0%)	315 (50.5%)	1261 (49.4%)	
District	Central	1348 (69.9%)	472 (75.6%)	1820 (71.3%)	0.021
	North	403 (20.9%)	104 (16.7%)	507 (19.9%)	
	South	178 (9.2%)	48 (7.7%)	226 (8.9%)	
Comorbidities	Deyo-Charlson co-morbidity index, mean (SD)	0.88 (1.55)	0.90 (1.47)	0.89 (1.53)	0.788
	Diabetes mellitus	207 (10.7%)	76 (12.2%)	283 (11.1%)	0.316
	Cardio-vascular disease	157 (8.1%)	65 (10.4%)	222 (8.7%)	0.079
	Hypertension	395 (20.5%)	157 (25.2%)	552 (21.6%)	0.014
	Chronic obstructive pulmonary disease	50 (2.6%)	21 (3.4%)	71 (2.8%)	0.307
	Cancer	141 (7.3%)	46 (7.4%)	187 (7.3%)	0.959
	Osteoporosis	108 (5.6%)	43 (6.9%)	151 (5.9%)	0.234
Other co-morbidities ^*^	Post-partum depression	5 (0.3%)	0 (0.0%)	5 (0.2%)	0.203
	Anxiety	460 (23.8%)	162 (26.0%)	622 (24.4%)	0.285
	Panic attacks	72 (3.7%)	16 (2.6%)	88 (3.4%)	0.241
	Personality disorder	103 (5.3%)	52 (8.3%)	155 (6.1%)	0.006
	Social phobia	16 (0.8%)	7 (1.1%)	23 (0.9%)	0.502
Smoking	Ever	926 (48.2%)	319 (51.1%)	1245 (48.9%)	0.324
	Never	995 (51.7%)	305 (48.9%)	1300 (51.0%)	
	Missing	2 (0.1%)	0 (0.0%)	2 (0.1%)	
Body mass index ^**^	Mean (SD)	26.54 (5.6)	26.66 (5.59)	26.57 (5.6)	0.635

^*^ within 1 year prior to index date

^**^ for those with a BMI measurement closest within 5 years before index date, *n* = 2374 (93.3%)

*TRD* treatment resistant depression

Factors associated with TRD included increasing age, suffering from personality disorder and not living in the northern region of the country (Table 2).

**Table 2:** Multivariable model (adjusted odds ratios) for factors associated with treatment resistant depression within one year from index date for the incident study cohort (patients with the start of any major depressive disorder episode within the study period, 1/1/2016–31/5/2018), *n* = 2553

		**Adjusted OR**	**95% CI**		***P*-value**
			**Lower**	**Upper**	
Age	per year	1.014	1.006	1.021	< 0.001
Sex	Female vs. Male	0.889	0.736	1.074	0.222
Socio-economic status	Low (ref.)				
	Medium	0.893	0.684	1.166	0.406
	High	0.939	0.754	1.169	0.572
District	Centre (ref.)				
	North	0.695	0.543	0.889	0.004
	South	0.758	0.535	1.074	0.119
Personality disorder	Yes vs. no	1.706	1.201	2.424	0.003

*CI* confidence intervals

**Fig. 4:**
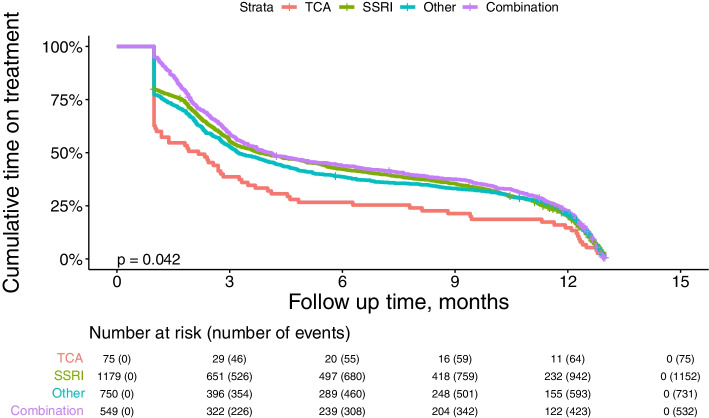
Time on treatment (months) on first-line treatment for patients in the incident study cohort (patients with the start of any major depressive disorder episode within the study period, 1/1/2016–31/5/2018), *n* = 2553. (Other monotherapy comprised: 39.1% venlafaxine, 21.5% duloxetine, 10.1% vortioxetine, 9.2% bupropion, 8.4% mirtazapine, 3.9% milnacipran). *TCA* tricyclic antidepressants, *SSRI* selective serotonin reuptake inhibitors

A total of 81.9% of patients purchased more than one month’s supply of treatment: 79.8% of those that purchased SSRI monotherapy, 95.1% of those that purchased combination therapy and 62.7% of those that purchased TCA monotherapy. Of those with TRD, 80.1% purchased more than one month's supply of treatment (as compared to 82.4% without TRD): 72.0% of those that purchased SSRI monotherapy, 96.2% of those that purchased combination therapy and 73.9% of those that purchased TCA monotherapy (Table 3). Median time on L1 treatment was 3.78 months for SSRI monotherapy, 4.11 months for combination therapy and 2.17 months for TCA monotherapy (Table 4, Fig. 4). For patients with no TRD, those that received L1 combination therapy or SSRI monotherapy had a longer median L1 time on treatment (9.50 [7.63, 10.65], 7.27 [6.08, 8.61] respectively) than those that received L1 other treatment or TCA (5.23 [4.24, 7.1], 2.25 [0.99, 8.12], respectively, *P* = 0.091). For patients with TRD, there was no difference in their time on treatment between different L1 therapies (*P* = 0.20).

**Table 3:** Medication purchases by type of antidepressant medication for one month or more, by treatment resistant depression, for the incident study cohort (patients with the start of any major depressive disorder episode within the study period, 1/1/2016–31/5/2018), *n* = 2553

		**Patients without TRD (*N* = 1929, 75.6%)**		**Patients with TRD (*N* = 624, 24.4%)**		**Total (*N* = 2553)**	
		N	%	N	%	N	%
**SSRI monotherapy**	Single purchase	163	17.9%	75	28.0%	238	20.2%
	> 1 purchase	748	82.1%	193	72.0%	941	79.8%
	**Total**	**911**	**100.0%**	**268**	**100.0%**	**1179**	**100.0%**
**Other monotherapy**	Single purchase	133	23.2%	37	20.9%	170	22.7%
	> 1 purchase	440	76.8%	140	79.1%	580	77.3%
	**Total**	**573**	**100.0%**	**177**	**100.0%**	**750**	**100.0%**
**Combination therapy**	Single purchase	21	5.3%	6	3.8%	27	4.9%
	> 1 purchase	372	94.7%	150	96.2%	522	95.1%
	**Total**	**393**	**100.0%**	**156**	**100.0%**	**549**	**100.0%**
**TCA monotherapy**	Single purchase	22	42.3%	6	26.1%	28	37.3%
	> 1 purchase	30	57.7%	17	73.9%	47	62.7%
	**Total**	**52**	**100.0%**	**23**	**100.0%**	**75**	**100.0%**
**Total**	Single purchase	339	17.6%	124	19.9%	463	18.1%
	> 1 purchase	1590	82.4%	500	80.1%	2090	81.9%
	**Total**	**1929**	**100.0%**	**624**	**100.0%**	**2553**	**100.0%**

(Other monotherapy comprised: 39.1% venlafaxine, 21.5% duloxetine, 10.1% vortioxetine, 9.2% bupropion, 8.4% mirtazapine, 3.9% milnacipran)

*TRD* treatment resistant depression, *TCA* tricyclic antidepressants, *SSRI* selective serotonin reuptake inhibitors

**Table 4:** Time on treatment (months) of L1 treatment using Kaplan–Meier analysis, for the incident study cohort (patients with the start of any major depressive disorder episode within the study period, 1/1/2016–31/5/2018), *n* = 2553

L1 treatment	TRD	N	Number (%) discontinued	Median time on treatment (95% CI), months	% on treatment at 3 months	% on treatment at 6 months	% on treatment at 12 months	Log rank P value
**SSRI monotherapy**	No TRD	911	884 (97.04%)	7.27 (6.08, 8.61)	65.2%	53.4%	25.7%	< 0.0001
	TRD	268	268 (100.00%)	2.04 (1.87, 2.27)	22.0%	4.5%	0.0%	
	**Total**	**1179**	**1152 (97.71%)**	**3.78 (3.32, 4.67)**	**55.3%**	**42.2%**	**19.8%**	
**Other monotherapy**	No TRD	573	554 (96.68%)	5.23 (4.24, 7.1)	60.4%	48.2%	27.2%	< 0.0001
	TRD	177	177 (100.00%)	2.07 (1.91, 2.33)	28.3%	7.9%	0.0%	
	**Total**	**750**	**731 (97.47%)**	**3.22 (2.96, 3.95)**	**52.8%**	**38.7%**	**20.8%**	
**Combination therapy**	No TRD	393	376 (95.67%)	9.50 (7.63, 10.65)	70.2%	58.7%	31.6%	< 0.0001
	TRD	156	156 (100.00%)	2.32 (2.01, 2.63)	30.1%	6.4%	0.0%	
	**Total**	**549**	**532 (96.90%)**	**4.11 (3.48, 5.26)**	**58.8%**	**43.8%**	**22.6%**	
**TCA monotherapy**	No TRD	52	52 (100.00%)	2.25 (0.99, 8.12)	42.3%	36.5%	21.2%	0.013
	TRD	23	23 (100.00%)	1.91 (1.18, 3.48)	30.4%	4.4%	0.0%	
	**Total**	**75**	**75 (100.00%)**	**2.17 (1.05, 3.32)**	**38.7%**	**26.7%**	**14.7%**	
**Overall**	No TRD	1929	1866 (96.73%)	6.97 (6.12, 8.19)	64.1%	52.5%	27.2%	< 0.0001
	TRD	624	624 (100.00%)	2.07 (1.97, 2.27)	26.1%	5.9%	0.0%	
	**Total**	**2553**	**2490 (97.53%)**	**3.65 (3.29, 3.98)**	**54.8%**	**41.1%**	**20.5%**	

(Other monotherapy comprised: 39.1% venlafaxine, 21.5% duloxetine, 10.1% vortioxetine, 9.2% bupropion, 8.4% mirtazapine, 3.9% milnacipran)

*TRD* treatment resistant depression, *SSRI* selective serotonin reuptake inhibitors, *TCA* tricyclic antidepressants

In the year after index, patients with TRD had an increased number of PCP visits, psychiatrist visits, ER visits, general hospitalizations and psychiatric hospitalizations (Table 5).

**Table 5:** Healthcare resource utilization for one year after index date for patients in the incident study cohort (patients with the start of any major depressive disorder episode within the study period, 1/1/2016–31/5/2018) by treatment resistant depression, *n* = 2553

		Patients without TRD (*N* = 1929, 75.6%)	Patients with TRD (*N* = 624, 24.4%)	Total *(n* = 2553)	*P*-value
Visits – Primary care physician	≥ 1, n (%)	1858 (96.3%)	613 (98.2%)	2471 (96.8%)	0.018
	Quantity, median (IQR)	10 (6, 16)	14 (9, 21)	11 (7, 17)	< 0.001
Visits—Psychiatrist	≥ 1, n (%)	1745 (90.5%)	600 (96.2%)	2345 (91.9%)	< 0.001
	Quantity, median (IQR)	3 (2, 5)	6 (3, 9)	4 (2, 6)	< 0.001
Emergency room visits	≥ 1, n (%)	415 (21.5%)	163 (26.1%)	578 (22.6%)	0.017
	Quantity, median (IQR)	1 (1, 2)	1 (1, 2)	1 (1, 2)	0.005
Hospitalizations	≥ 1, n (%)	262 (13.6%)	138 (22.1%)	400 (15.7%)	< 0.001
	Number of separate hospitalizations, median (IQR)	1 (1, 2)	1 (1, 2)	1 (1, 2)	0.267
	Number of nights, *median (IQR)*	4 (2, 13.5)	6 (2, 39)	5 (2, 20)	0.031
Psychiatric Hospitalizations	≥ 1, n (%)	66 (3.4%)	57 (9.1%)	123 (4.8%)	< 0.001
	Number of separate hospitalizations, median (IQR)	1 (1, 1)	1 (1, 2)	1 (1, 2)	0.031
	Number of nights, *median (IQR)*	20 (6, 42)	40 (10, 67)	27.5 (8, 61)	0.279
Electroconvulsive therapy	≥ 1, n (%) ^a^	14 (0.7%)	18 (2.9%)	32 (1.3%)	< 0.001
	Time to ECT treatment, *for those that initiated treatment after index date*, median (IQR)	5.39 (3.12, 18.63)	10.5 (4.28, 16.95)	9.99 (3.53, 16.25)	0.733

^a^excluding those with ECT before index date

*TRD* treatment resistant depression, *ECT* electroconvulsive therapy

A sub-analysis performed on a cohort of patients with a first ever MDD episode showed similar results.

## Discussion

### Period prevalence cohort

Major depressive disorder is a severe disorder that had an average global prevalence in 2010 of 6% [2], with a recent systemic review reporting lifetime prevalence of between 2 and 21% [36]. Approximately 6.7% of adults over the age of 18 had a major depressive episode in the US in 2015 [37, 38] which increased to 8.4% in 2020 [39]. Prevalence rate found in our study was very low compared to published rates around the world [40] and also compared to the World Health Organization World Mental Health Study which found a prevalence in Israel of 5.9% [41]. In 2007 a process to transfer responsibility of mental health services from the Ministry of Health to the health funds (MHS is one of four health funds in Israel) was initiated and took effect in July 2015. Therefore the low prevalence rate found in our study could be due to the fact that even though the responsibility for mental health patients passed to the health funds, many patients with MDD were still treated in out-patient clinics of psychiatric hospitals (not associated with the health funds), and their diagnoses did not reach the MHS health fund's databases during our study period. In addition, this low rate found in the MHS database could be due to under reporting and under diagnosis by physicians. A literature review on depression diagnosis in primary care in Israel described the challenges that need to be overcome in order to provide better care to these patients [42]. The authors describe a prevalence of MDD of 1.6–5.9%, associated with female sex and fewer years of education. They describe how many cases were undiagnosed and how most patients had persistent depression or achieved only partial remission. The Israeli population consists of a rich variety of cultural backgrounds, beliefs and languages, and many immigrant populations. Immigrants are known to be at high risk of depression [43] and communication limitations may make diagnosis and treatment challenging. In addition, the stigma of mental illness is still high in Israel and patients may convince their physicians not to report a major depressive diagnosis in the electronic database [44], or report less a severe disease diagnosis such as anxiety. The process of transfer of responsibility of mental health services from the Ministry of Health to the health funds increased the number of patients with MDD that primary care doctors needed to diagnosis and treat, therefore necessitating specialist knowledge and timely referral to a psychiatrist.

Among patients diagnosed with MDD, nearly one quarter of the incident MDD cases were TRD in line with previous studies [17, 25, 45, 46]. Another study reported a much higher proportion of TRD, however this was a clinical trial where all patients received medication according to protocol [8]. TRD increased with age and ranged between 11.4% for under 18 year olds to 28.3% for patients aged over 65 years old. In real-world clinics, not all patients will receive treatment or move to another line of therapy, however we found that untreated patients made up just 3–20% of the entire prevalence cohort (depending on age group and sex), lower than observed in other countries [41]. This highlights that although the prevalence observed in Israel is lower than other countries, once diagnosed, patients are likely to receive treatment.

### Incidence cohort: Treatment patterns

Median age in the incidence cohort at index date was 47 years (IQR 36–56), similar to age reported in another retrospective database study [47].

We found a higher prevalence of MDD amongst women across all age groups, with almost twice as many women than men, confirmed by previous studies [5, 48]. It has been suggested that women present with more depressive symptoms than men, who less frequently meet the diagnostic threshold for a MDD diagnosis. Another theory proposed that men and women have different types of symptoms, with men ascribing depression to work related issues and women ascribing depression to relationship problems. This theory also highlights different coping mechanisms with men pursuing sports or drinking alcohol and women using emotional outlets [49]. However, many structural changes have been taking place over the last few decades as more women join the workforce and share childcare responsibilities, which may influence prevalence of MDD. Another study reports how socioeconomic and family related factors significantly effect this variation between the sexes, with lower risk of depression associated with marriage or cohabiting with a partner and with higher socioeconomic level [50].

Our analysis indicates that TRD was significantly associated with personality disorder. Personality disorder may present as depressive mood, showing an interaction between the two disorders [51, 52] and is associated with poor response to treatment [53, 54]. Previous studies have shown that improvement in MDD affects the outcome of personality disorder [55]. Other factors associated with TRD include older age, marital status, long duration of current MDD episode, anxiety, higher suicidal risk and high numbers of hospitalization [56]. Residential area was an unexpected factor associated with TRD and is relevant only in the Israeli setting. We suggest that this finding may reflect healthcare disparities since this region has less access to healthcare including mental health professionals and thereby leading to under diagnosis.

SSRIs were the most frequently initiated L1 treatment (46%). A total of 22% received combination therapy, (consisting of any combination of at least two antidepressants drugs: 51% SSRI + another antidepressant drug, and 19% combination of two other antidepressants). Whereas there are no clinical trials that recommend combination therapy and the increased effectiveness is debated [57–60], there is some evidence to suggest that combination therapy may be effective particularly in the elderly [61], and the combination of venlafaxine and mirtazapine may be particularly effective in difficult-to-treat depression [62, 63]. Advantages of combination therapy may include rapid response with no titration necessary, however may have disadvantages of adverse reactions and adherence issues.

Our study shows that median time on L1 treatment was 3.6 months and 18% had a single medication purchase only. Guidelines recommend continuing antidepressants for 4 to 9 months after initial symptom resolution, however, many patients discontinue earlier (most within first 3 months) probably due to side effects, lack of efficacy or improvement of symptoms [64]. TCA treatment was more likely to be discontinued after one month, in line with previous studies that report increased side effects with this therapeutic group [65] and patients that initiated SSRI or combination therapy for L1 with no TRD had longer time on treatment than those that received TCA or other treatment.

Patients with TRD had increased healthcare utilization including PCP and psychiatry visits, hospitalizations and ER visits, incurring higher burden of disease and healthcare costs as reported in previous studies [46, 66]. A previous study has found that patients suffering from TRD have greater risk of unemployment, reduced work productivity, and poorer patient health-related quality of life compared to responders [67]. To date, treatment options have been limited, however the treatment landscape is evolving, and a novel agent esketamine has been approved by the FDA and the Israel Ministry of Health in the last year for patients with MDD and TRD after failure of two previous lines of treatment, allowing for an additional therapeutic approach for these patients.

The strengths of this study include the overall size of the sample and the real-world generalizability of data drawn from a broad claims database in Israel. However, it may have limitations associated with its retrospective cohort design. Data on purchases made outside of MHS pharmacies were not captured; however, patients are unlikely to buy medications outside of MHS due to their discounted price within MHS. It should also be noted that actual medication use is unknown, as dispensed medications may not be consumed. However, previous studies have demonstrated the validity of this approach for measuring compliance with chronic medications [68].

## Conclusion

Our study shows that prevalence of MDD in Israel is low compared to other countries, however once diagnosed patients are likely to receive drug treatment. Among patients diagnosed with MDD, the proportion of TRD is similar to other countries, increases with age and is associated with increased healthcare utilization, therefore should be a focus of continued research for finding effective long term treatment options. 

## Data Availability

The datasets generated and analyzed during the current study are not publicly available due to restrictions in MHS and the Israel Ministry of Health but are available from the corresponding author on reasonable request.
